# Left ventricular function, morphology, and myocardial tissue characterization in Sickle Cell Disease: a multi-modality imaging study

**DOI:** 10.1186/1532-429X-13-S1-P334

**Published:** 2011-02-02

**Authors:** Ahmad Homaa, Ankit A Desai, Roberto M Lang, Thejasvi Thiruvoipati, Kristen Turner, Lynn Weinert, E Bruce Jamison, Nicole Artz, Sharon Trevino, Sharon Feehan, Roberto Machado, Joe GN Garcia, Amit R Patel

**Affiliations:** 1 University of Chicago Medical Center, Chicago, IL, USA; 2 Univeristy of Illinois at Chicago, Chicago, IL, USA; 3 Loyola Medical Center, Chicago, IL, USA

## Objectives

Our aim was to characterize the cardiac morphology, function and myocardium in patients with sickle cell disease (SCD) using CMR and transthoracic echocardiography (TTE).

## Background

Cardiovascular complications are a major cause of death in SCD yet the mechanism remains unclear.

## Methods

Thirty-one stable, African-American outpatients with SCD (mean age 32±8 yrs) prospectively underwent CMR (Philips 1.5 Tesla) and TTE (Philips iE33). Retrospectively-gated cines of left ventricular (LV) 2-, 3-, and 4-chamber, and short axis cine stack were obtained using SSFP (temporal resolution 25-40ms). Late gadolinium enhancement (LGE) images of the same views were obtained 10-20 minutes after infusion of Gd-DTPA (0.15 mmol/kg) using phase sensitive inversion recovery (TR 4.5 ms, TE 2.2ms, TI 250-300 ms, flip angle 30°, PSIR flip angle 5°).

Single short-axis, mid-ventricular myocardial T2* slice and coronal, hepatic T2* slice were acquired with a single breath-hold, at six echo-times (2.3 to 14 msec) using a gradient echo sequence. Tissue T2* signal intensity was measured in LV septum and liver at two separate echo times and T2*= - ΔTE/ln (SI_TE2_/ SI_TE1_) where ΔTE represents time difference between the two echo times and I_TE1_ and I_TE2_ represent signal intensity at echo time one and two. Myocardial and hepatic T2* were abnormal if <20ms and <18ms, respectively.

CMR LV volumes, ejection fraction (EF), and mass were calculated using method of disks and indexed for body surface area. The presence or absence of LGE was determined. Diastolic dysfunction (DD) was identified based on echocardiographic measurements including tissue Doppler (age adjusted E/A ratio) and left atrial volumes.

## Results

**Figure 1:**
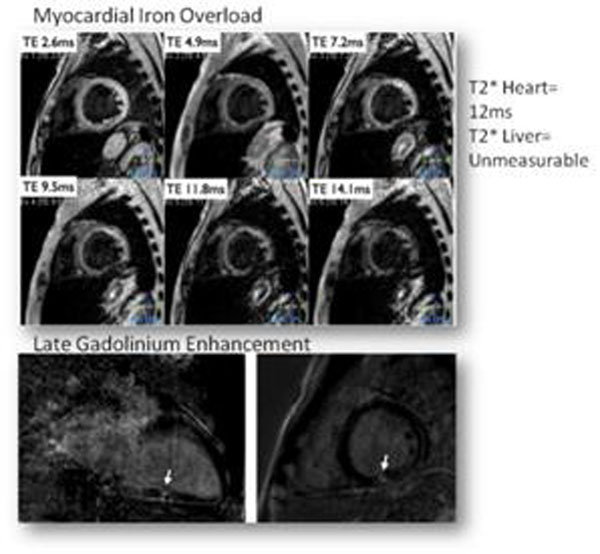


SCD patients had preserved LVEF with enlarged biventricular and LA volumes. DD was present in 15 (48%). LGE was noted in 7 (23%), myocardial iron overload in 2 (7%) and hepatic iron overload in 16 (52%). (Figure 1) Those with LGE had significantly lower hepatic T2* (p<0.01) and a trend towards having a larger LVEDV index, LV mass index, RVEDV index, and LA volumes. (Table 1) Woman with LGE (versus women without LGE) had significantly larger LVEDV index 133.4±9 versus 108.4±25 (p= 0.01), RVEDV 230.3±13 versus 193.9±41 (p= 0.02), and LA volume 146.7±15 versus 94.2±20 (p=0.01).

**Table 1:** 

	All Study Patients (n=31)	No LGE (n=24)	Positive LGE (n=7)	p-value
LV EF (%)	58.3 ± 7	58.4 ± 5	58.9 ± 12	0.92
LVEDV (mL)	224.8 ± 61	217 ± 57	251.1 ± 62	0.25
LVEDV Index (mL/m^2^)	124.2 ± 29	118.9 ± 27	142.6 ± 25	0.07
LV mass (g)	141.1 ± 40	135.6 38	159.9 ± 36	0.18
LV mass Index (g/m^2^)	78.1 ± 20	74.3 ± 18	91.3 ± 17	0.06
RVEDV (mL)	227.2 ± 59	222.3 ± 60	244.3 ± 50	0.38
RVEDV Index (mL/m^2^)	125.5 ± 27	121.4 ± 27	139.4 ± 22	0.12
LA volume (mL)	117.1 ± 34	112.2 ± 33	134 ± 26	0.11
T2* Myocardial Iron (msec)	42.2 ± 13	45 ± 11	34 ± 16	0.18
T2* Hepatic Iton (msec)	17.9 ± 14	21.7 ± 13	4.9 ± 3	<0.01

## Conclusion

SCD patients had significant chamber dilation, DD, and LGE. Those with LGE had significantly more hepatic iron overload suggesting a greater disease severity requiring more blood transfusions. Women with LGE demonstrated more severe adverse remodeling. Myocardial iron overload was rare.

